# Hemorrhage in Abortions

**Published:** 1854-09

**Authors:** 


*Hemorrhage in Abortions.*—Cases of abortion are frequently met with in which there continues a tedious, debilitating, and (to the patient at least) alarming hemorrhage, after the ovum is supposed to have passed. The young practitioner may conclude that the placenta, as well as the fœtus, has been discharged either by relying upon the information of attendants, or by having himself seen what he supposed was the whole afterbirth. I have never yet, however, that I am aware of, met with a case in which there was, in the earlier months, any hemorrhage after a clear delivery of the ovum. In every instance in which it continued after the supposed delivery, I have ascertained that a piece or pieces of the placenta or a portion of membrane had been retained.

In these cases I have found medicines of very little use; indeed, often more inconvenient than useful. Generally the mouth, or perhaps more properly speaking, the *neck* of the womb is too much contracted to allow the introduction of more than one finger, and frequently too much so for that. If a finger can be introduced, the placenta, or a portion of it, may be found presenting, or it may not be felt. When it can be reached by the finger, it may often be drawn out, a piece at a time, by hooking the finger around it. When it could not be delivered in this way, I have known astringents, opiates, cold water, ergot, &c. to be used in vain. Any effect they had was merely temporary. Dewees' hook would in such cases be generally unavailable, as it could not take sufficient hold to overcome the resistance of the womb, and would, therefore, tear out. Nor am I disposed to think much more highly of Doctor Bond's forceps; as it seems to me that whenever they could be employed, the object would be more safely and easily effected by carefully introducing the hand into the vagina, and one or two fingers into the womb. Such has been my practice.

When the delivery cannot be safely and readily effected by manual assistance, I should pronounce the tampon the appropriate remedy. It arrests the hemorrhage; and, by causing a certain amount of blood to accumulate and coagulate within the uterus, it seems to facilitate its healthy and efficient action. In a day or two after its introduction, I have uniformly found the offending cause (whether it were the afterbirth, or a portion of it only,) lying in the vagina, or in the relaxed mouth of the womb; or it had been expelled together with the tampon, by a strong uterine effort, or whilst the patient was on the night vessel.

A few cases will be briefly given in illustration of these views:

Several years ago the writer was called in consultation to a case under the care of a medical friend, who stated that about a week before, the patient, a delicate lady, had miscarried at the third month; and that she had had more or less hemorrhage ever since, and was then becoming very weak. He had used various remedies in his efforts to arrest it, but in vain. He was asked if the afterbirth had passed. He said yes, that he had seen it himself, and felt assured that the whole of it had come away. He did not think he could be mistaken about it, that the patient was in an erect posture when it dropped from her. He was so positive as to the correctness of his observations, that I felt a delicacy in proposing an examination; and contented myself with suggesting some additional medicines, cold water, &c. These were faithfully administered, but their effect was merely temporary. On my next visit I expressed the opinion that possibly, as the lady was in an erect posture when the afterbirth was passed, the womb may have grasped a portion of it, from which the rest broke away by its weight in falling; and therefore proposed an examination. The os uteri was found soft, bathed in blood, and patulous; but the neck was contracted, so that the forefinger could be only partially introduced. Some pieces of placenta, in a putrid condition, were found presenting, which were with difficulty hooked out. Others were felt that could not be brought away. The tampon was then applied, and the next day nearly all of the remaining portions, in very small pieces, were taken away, several of them being found in the vagina. The patient, though better now, was not entirely relieved until the day after, when a few more crumbs were taken away, and the hemorrhage ceased. What struck me forcibly in this case, and some others that I had previously attended, was, that until the last portion of placenta was passed, the os uteri continues soft and patulous, with blood continually oozing therefrom; but when that had occurred, it immediately became firm, closed, and comparatively dry.

In the course of the last year I was called to a woman who had miscarried after the fourth month. She was suffering with violent pains, accompanied with considerable hemorrhage. Herself and her attendant thought the secundines had been discharged, and upon making an examination I could feel nothing presenting. As her general health was bad, it was thought possible that the hemorrhage was kept up from that cause, and such medicines as appeared to be indicated were prescribed. The uterine symptoms were no better the next day, which confirmed a suspicion that a portion (at least of afterbirth) was retained. The tampon was then applied, an anodyne administered, and the patient assured that she would soon be relieved. In the course of the following night the tampon and a large piece of placenta incased in clotted blood were thrust out by one strong effort of the womb, and immediate relief of all the symptoms followed.

During the last month a lady who had been pregnant just four weeks, was taken, after some extraordinary fatigue, with quite an abundant flow from the uterus. Though this was out of time by one week, and preceded by feelings of unusual discomfort, she still thought it was only a return of her menses. It continued pretty free for about five days, when there escaped from her whilst on the night vessel an ovum a little over an inch long, perfectly white, excepting a spot at one end, and covered externally with the chorion. As this was something she did not understand, it was shown to me. The nature of the case was explained to her; and as the hemorrhage still kept up, I informed her that I presumed there was a membrane (the decidua) to be discharged, and when that happened she would no doubt be relieved. I begged her, at the same time, carefully to watch for it, and to show it to me when it had passed. The next morning about day it was discharged, and the hemorrhage ceased entirely. This case was not deemed serious enough to call for the use of the tampon; but is considered strikingly to sustain the position, that when the flow continues in abortions of the earlier months, it is an evidence that some portion of the secundines is yet to be passed.

Now, though the result of my experience is as above stated, it would be rash in me to lay it down as an invariable rule; neither is it pretended that there will be hemorrhage in every case in which there is retention. In this latter respect there is a marked difference in different patients, as is still more strikingly displayed in labors at the full time. At this period the wombs of some persons will commence flooding as soon as the afterbirth is detached, and even after it is delivered, will continue to pour out blood in a dangerous quantity unless kept in the *firmest* state of contraction for a day or two afterwards. In the majority of cases, however, when an individual has had two or more children, the uterus will after delivery alternately expand and contract without any other effect than the usual afterpains and lochial discharge. The practitioner observing this, will not think it necessary in all cases to observe the rule of Dr. Dewees, which requires that he shall not leave the bed side of his patient until the uterus is permanently contracted. A prudent man, however, will satisfy himself well upon these points before leaving his patients. P.

*Stethoscope.*

